# Sexual Risk Factors for HIV Infection in Early and Advanced HIV Epidemics in Sub-Saharan Africa: Systematic Overview of 68 Epidemiological Studies

**DOI:** 10.1371/journal.pone.0001001

**Published:** 2007-10-03

**Authors:** Li Chen, Prabhat Jha, Bridget Stirling, Sema K. Sgaier, Tina Daid, Rupert Kaul, Nico Nagelkerke, for the International Studies of HIV/AIDS (ISHA) Investigators

**Affiliations:** 1 Centre for Global Health Research, St. Michael's Hospital, University of Toronto, Toronto, Ontario, Canada; 2 Division of Medical Sciences, Island Medical Program, University of Victoria, Victoria, British Columbia, Canada; 3 Department of Medical Microbiology, University of Nairobi, Nairobi, Kenya; 4 Department of Medicine, University Health Network, University of Toronto, Toronto, Ontario, Canada; 5 Department of Community Medicine, Al Ain University, Al Ain, United Arab Emirates; Medical Research Council South Africa, South Africa

## Abstract

**Background:**

It is commonly assumed that sexual risk factors for heterosexual HIV transmission in sub-Saharan Africa, such as multi-partner sex, paid sex and co-infections, become less important as HIV epidemics mature and prevalence increases.

**Methods and Findings:**

We conducted a systematic review of 68 African epidemiological studies from 1986 to 2006 involving 17,000 HIV positive adults and 73,000 controls. We used random-effects methods and stratified results by gender, time, background HIV prevalence rates and other variables. The number of sex partners, history of paid sex, and infection with herpes simplex virus (HSV-2) or other sexually-transmitted infections (STIs) each showed significant associations with HIV infection. Among the general population, the odds ratio (OR) of HIV infection for women reporting 3+ sex partners versus 0–2 was 3.64 (95%CI [2.87–4.62]), with similar risks for men. About 9% of infected women reported ever having been paid for sex, versus 4% of control women (OR = 2.29, [1.45–3.62]). About 31% of infected men reported ever paying for sex versus 18% of uninfected men (OR = 1.75, [1.30–2.36]). HSV-2 infection carried the largest risk of HIV infection: OR = 4.62, [2.85–7.47] in women, and OR = 6.97, [4.68–10.38] in men. These risks changed little over time and stratification by lower and higher HIV background prevalence showed that risk ratios for most variables were larger in high prevalence settings. Among uninfected controls, the male-female differences in the number of sex partners and in paid sex were more extreme in the higher HIV prevalence settings than in the lower prevalence settings.

**Significance:**

Multi-partner sex, paid sex, STIs and HSV-2 infection are as important to HIV transmission in advanced as in early HIV epidemics. Even in high prevalence settings, prevention among people with high rates of partner change, such as female sex workers and their male clients, is likely to reduce transmission overall.

## Introduction

Sub-Saharan Africa has only 10% of the world's population, yet it accounts for about 60% of the estimated 40 million HIV infections globally [1]. As of December 2006, 37 of 43 sub-Saharan African countries, and 15 of 18 countries in Eastern and Southern Africa, were categorized by UNAIDS as having a “generalized epidemic”, defined as an HIV prevalence of more than 1% in the adult general population (aged 15–59) and more than 5% in adult vulnerable groups, such as patients of sexually-transmitted infection (STI) clinics. The key risk factors for heterosexual transmission (the dominant mode of acquiring HIV in Africa) are transactional or paid sex, concurrent partners [2]–[3], co-infection with viral and bacterial STIs, notably herpes simplex virus type 2 (HSV-2) [4], and lack of male circumcision [5]–[6]. Mathematical modeling [7] and several epidemiological studies [2]–[3] find that vulnerable groups such as female sex workers (FSW) and their clients, who have high rates of acquiring and transmitting HIV, play key roles in the spread of HIV and for maintaining HIV infection levels in the general population [8].

Conventional wisdom (abetted by the UNAIDS definition of generalized epidemics) by contrast has argued that multi-partner sex, paid sex and STIs are important risk factors during the early stages of the epidemic, when infections are concentrated in high-risk groups, but matter less in advanced epidemics when a much larger proportion of “low-risk” individuals is infected. If this wisdom were true, we would expect the risks of HIV from high-risk sexual behavior to diminish over time as HIV prevalence increases. If true, this would also imply that high coverage of condom programs among FSWs and their clients would not stop high levels of HIV transmission, and prevention programs would thus need to focus as well on reducing sexual exposure within marriage and similar relationships [9].

There are few robust epidemiological studies in Africa that could help resolve this controversy. In their absence, we conducted a systematic review of 68 epidemiological studies in order to quantify individual risks for sexual risk factors, assess if these risks have changed over time, and to examine if the strength of these associations varies by background prevalence of HIV infection.

## Methods

### Literature search

We conducted a computerized search of Embase, Medline, PRE-MED, and Web of Science with the keywords: ‘HIV’, ‘HIV-1’or ‘delta retrovirus’, ‘horizontal transmission’, ‘risk factor’, ‘sexually-transmitted infections or disease’, ‘herpes’ or ‘HSV’, and ‘Africa’ (exploded to include countries within Africa). We excluded studies of male circumcision because there is little doubt about its protective effect in high prevalence settings [10]. Also, earlier reviews have shown that the magnitude of risk reductions afforded by male circumcision have changed little over time in high-prevalence settings and the protective effect of circumcision is, if anything, stronger among high-risk men than among low-risk men [5]–[6]. Studies were limited to those published in English or French after 1986 and before June 2006. All computerized hits were examined by one of two investigators (LC, BS or TD) to select eligible abstracts. Two investigators read the articles independently to examine if studies met the inclusion criteria, and then excluded those of poor quality, those of inadequate size, and those that included non-pertinent populations. No efforts were made to include unpublished studies or conference abstracts because of their inadequate details. Inclusion criteria were based on whether the study provided sufficient information on demographic characteristics such as country, rural or urban location, characteristics of the population, study design variables, such as clear definitions of sexual risk factors, methods of diagnosing STI (as self-reported or clinical examination), number of cases and controls, and crude risk and adjusted risk estimates with 95% confidence interval (95% CI). Details of each study [11]–[113] were entered into a database by one investigator with a 100% re-check. A quality assessment was carried out using a standard operating procedure [6]. This procedure assessed misclassification of exposures, outcomes, and confounders (age, residence, religion, education, marital status, sexual behavior, condom use, any STI, male's circumcision status), as well as representativeness of samples (high-risk groups, general population or other).

### Case and controls

Cases were defined as HIV seropositive participants, and controls as all non-infected participants drawn from the same populations as cases (irrespective of study design). Testing protocols varied among studies but generally followed WHO's recommendations of two positive ELISA tests [114].

### Exposures

Number of sex partners was grouped on the basis of lifetime partners or, if unavailable, on the basis of partners during the previous 5 or more years, as: low (2 or more versus 0–1); high (3 or more versus 0–2); or higher (4 or 5 or higher versus 0–2). Paid sex for women was defined as having ever been a FSW, and for men as having ever been a client of a FSW. STI history was defined as a clinical history of chancroid, herpes, syphilis, genital warts, chlamydia, gonorrhea or trichomoniasis confirmed by laboratory testing or clinical examination. HSV-2 infection was based on antibody assay or by clinical examination.

Studies were stratified by the expected risk of their target populations (classified as either high-risk or general) sexual behavior and lifestyle. High-risk population studies mainly included truck drivers, FSWs, hotel and bar workers and STI clinics attendees. General populations included pregnant women, factory workers, and patients in general hospitals. Sensitivity of results to stratification criteria was explored by excluding factory workers and hospital patients, which yielded results similar to those presented here (data not shown). We reclassified 4 studies [13], [35], [45], [52] with unusually high levels of risk factors among their controls from general to high-risk population studies.

### Stratification by time and background HIV prevalence

Studies were stratified by year, as defined by the midpoint of the study period. If study year was unavailable we used the year prior to publication. We assigned background rates of HIV prevalence based on UNAIDS data [1] for the study year or the year closest to it, stratified by urban or rural status (defined by the location of the study or the residence status for the majority of the study participants). Studies were then grouped into “lower” (< = 20%) or “higher” (>20%) background prevalence, based on the medians observed (use of a cut-off of 10% prevalence gave very similar results, results not shown).

### Statistical methods

We calculated the crude odds ratios (OR) to estimate the strength of risk factors for HIV-1 infection for each study. Due to the apparent high heterogeneity among the studies, ORs were calculated using the random effects method of DerSimonian & Laird [115]. Heterogeneity was tested by chi-square. Tests for trend used the method of weighted regression [116] and tests for differences in summary ORs used the Tarone method [117]. Begg's test [118] was used to detect publication bias. All statistical analyses were done in STATA, version 8.0.

## Results

Our computerized search generated 1,204 hits, which after a single reading by one of two investigators (LC, BS or TD) yielded 550 abstracts. Independent reading by two investigators yielded 110 papers [11]–[113] meeting these criteria. Of these 110 papers, a further 36 [78]–[113] were excluded for various reasons, such as non-pertinent partnerships (discordant couples [110]–[112] or other irrelevant partnerships [50], [82], [109]). To avoid unstable estimates and minimize publication bias, 8 studies that provided less than 60 people overall or less than 20 cases or controls were excluded: five of paid sex: [106]–[110]; five of STI [84], [108]–[111]; and one of HSV-2 [50]. In total 68 studies (from 67 papers) from 18 countries with a total population of 17,000 HIV positive adults and 73,000 controls were included: 62 studies used cross-sectional or case-control design and 6 used longitudinal designs. Table S1 provides the characteristics of the studies, including sample size, year, study design, and by source of controls (general population or high-risk).

### Risk factors among the general population

**Figure 1:**
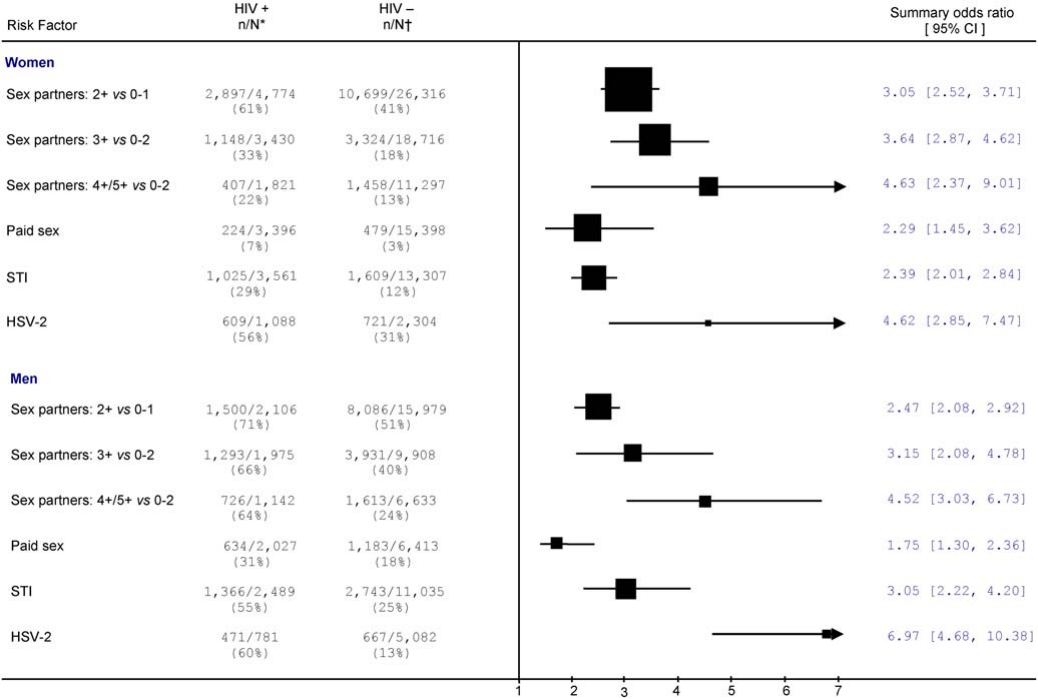
Summary odds ratios of risk factors for HIV among the general population for women and men. Notes: * n/N represents the number of HIV-positive individuals with exposure to the risk factor over the total number of HIV-positive individuals. † n/N represents the number of HIV-negative individuals with exposure to the risk factor over total number of HIV-negative individuals. The size of the square is proportional to the size of the total population

Figure 1 summarizes our key findings from general population studies, totaling 14,000 cases and 65,000 controls. There was a clear relationship with the number of lifetime sex partners in women and men. For example 61% of HIV positive women reported 2 or more sexual partners versus only 41% of uninfected control women (OR = 3.05 (95%CI [hereafter in square parentheses; [2.52–3.71]). Odds ratios increased with (higher) number of reported partners: about 33% of HIV positive women and 18% of uninfected controls reported 3 or more partners (OR = 3.64, [2.87–4.62]), while 22% of cases and 13% of controls reported 4 or more partners (OR = 4.63, [2.37–9.01]. This increase in ORs was not statistically significant (test for trend = 0.09). Similarly elevated risks were seen for men with higher number of partners: men reporting 3 or more partners versus 0–2 had an OR of 3.15 [2.08–4.78]) and men with 4 or 5 partners further increased their risk (although not-significantly; test for trend = 0.11). About 7% of infected women reported ever having been paid for sex versus 3% of control women (OR = 2.29, [1.45–3.62]). About 31% of HIV infected men reported ever having sex with a FSW, as against 18% of control men (OR = 1.75, [1.30–2.36]). HSV-2 was the STI most strongly associated with HIV in women (OR = 4.62, [2.85–7.47]) and in men (OR = 6.97, [4.68–10.38]). Lower risks were seen for any history of STIs among men and women.

### Variation in risk over time

**Figure 2:**
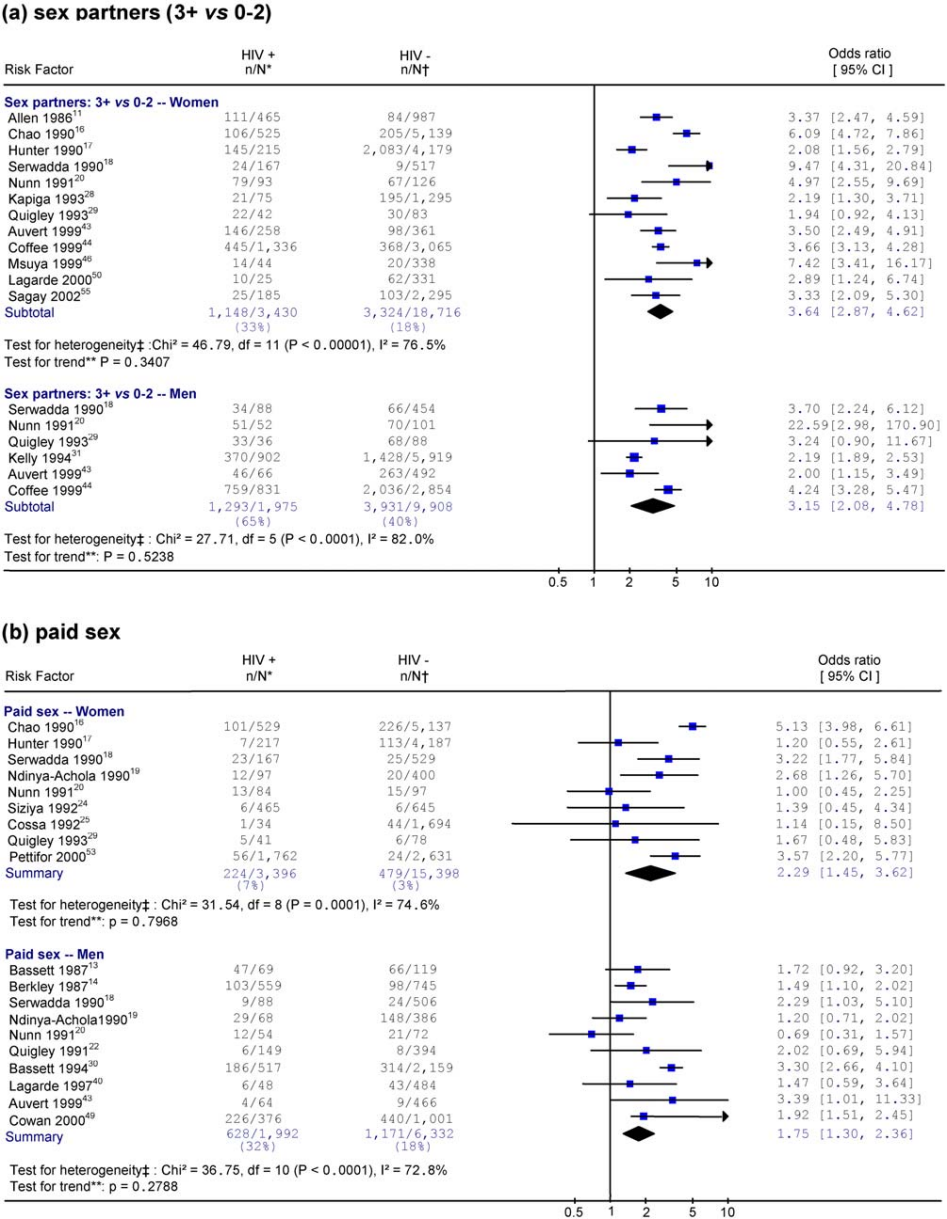
Studies of HIV and selected risk factors in Africa. Notes: * n/N represents the number of HIV-positive individuals with exposure to the risk factor over the total number of HIV-positive individuals. † n/N represents the number of HIV-negative individuals with exposure to the risk factor over the total number of HIV-negative individuals. ‡ Heterogeneity is tested by chi-square (p<0.05 indicates a significant heterogeneity). I-squared reflects the variation in summary OR attributable to heterogeneity among studies. ** Weighted linear regression method is used to test the linear trend of ORs by the study year (p<0.05 indicates a significant trend). Each risk factor is listed by order of the study year. The size of the square is proportional to the weight of the respective OR. Different scales are used for HSV-2 studies

**Figure 3:**
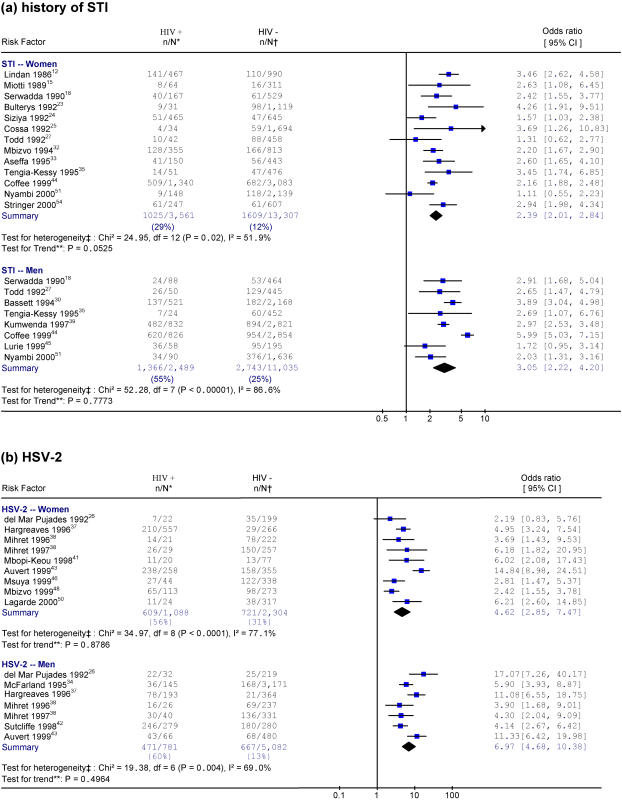
Studies of HIV and selected risk factors in Africa. Notes: * n/N represents the number of HIV-positive individuals with exposure to the risk factor over the total number of HIV-positive individuals. † n/N represents the number of HIV-negative individuals with exposure to the risk factor over the total number of HIV-negative individuals. ‡ Heterogeneity is tested by chi-square (p<0.05 indicates a significant heterogeneity). I-squared reflects the variation in summary OR attributable to heterogeneity among studies. ** Weighted linear regression method is used to test the linear trend of ORs by the study year (p<0.05 indicates a significant trend). Each risk factor is listed by order of the study year. The size of the square is proportional to the weight of the respective OR. Different scales are used for HSV-2 studies

Figure 2 and 3 provide the specific studies in chronological order for four exposures among the general population: 3 or more sex partners versus 0–2, being an FSW or a male FSW client, STI history, and HSV-2 infection. There were no differences between early and late time periods in the strength of the association between all these exposures and HIV and all tests for time trend were non-significant.

### Variation in risks by background prevalence of HIV

**Figure 4:**
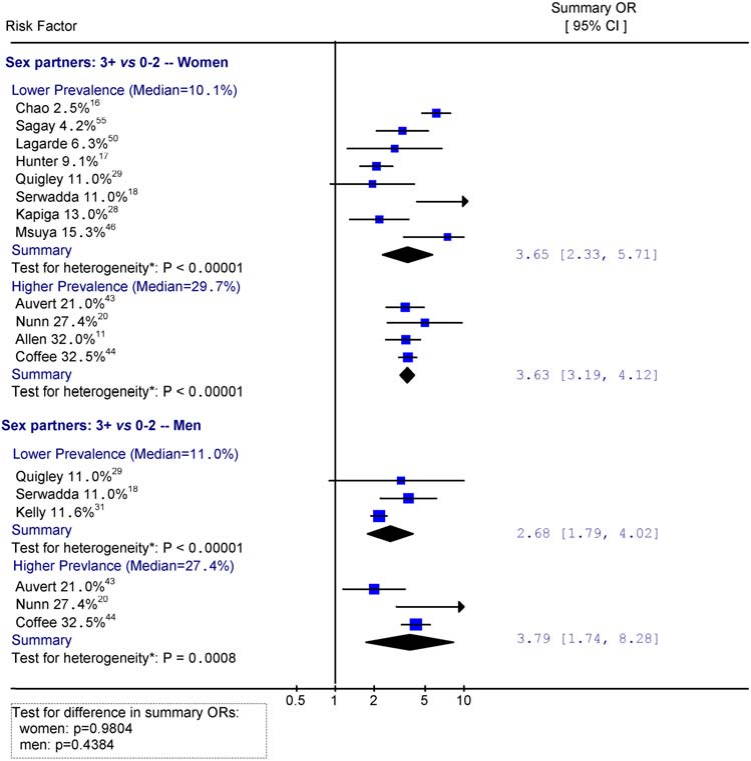
Sex partners (3+ vs. 0–2) by lower and higher background HIV prevalence. Notes: * Heterogeneity is tested by chi-square (p<0.05 indicates a significant heterogeneity). The size of the square is proportional to the weight of the respective OR. Different scales are used for HSV-2 studies

**Figure 5:**
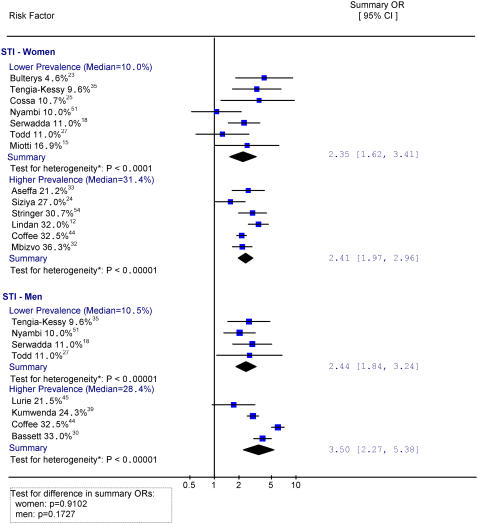
History of STI by lower and higher background HIV prevalence. Notes: * Heterogeneity is tested by chi-square (p<0.05 indicates a significant heterogeneity). The size of the square is proportional to the weight of the respective OR. Different scales are used for HSV-2 studies

**Figure 6:**
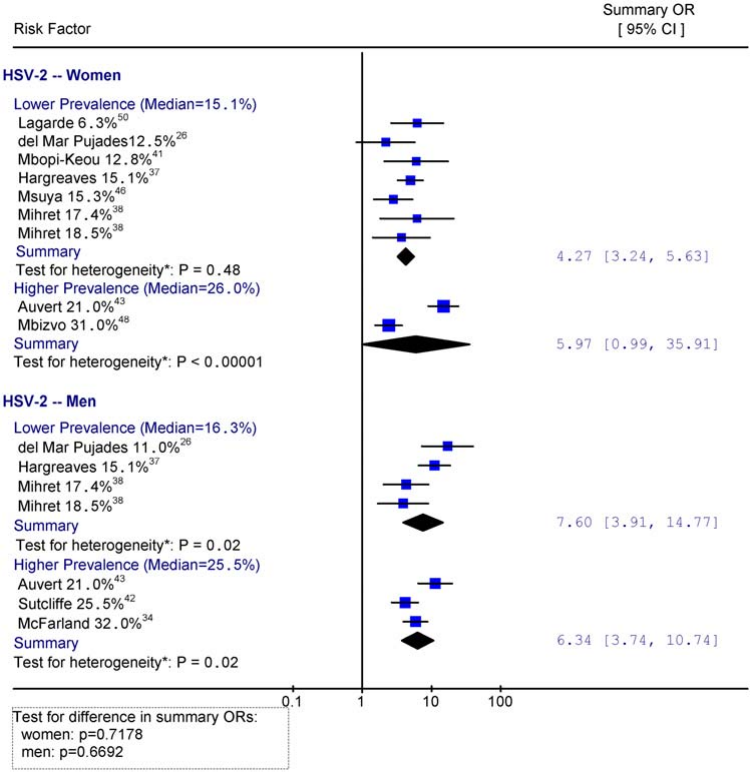
HSV-2by lower and higher background HIV prevalence. Notes: * Heterogeneity is tested by chi-square (p<0.05 indicates a significant heterogeneity). The size of the square is proportional to the weight of the respective OR. Different scales are used for HSV-2 studies

Figure 4, 5 and 6 provide results for studies documenting 3 or more sex partners versus 0–2, history of STI, and HSV-2 infection, grouped by lower and higher antenatal clinic prevalence (as a proxy for background prevalence). While the analyses used a 20% prevalence to delineate the comparison groups, the actual mean background prevalence varied as shown. For women, there were no significant differences in risk of HIV infection with 3 or more sex partners versus 0–2 between lower (OR = 3.65) and higher (OR = 3.63) background HIV prevalence. Similarly, there were few differences in risk of HIV with STI history between lower (OR = 2.35) and higher (OR = 2.41) background HIV prevalence. Finally, there was no major difference in risk of HIV with HSV-2 infection between lower (OR = 4.27) and higher (OR = 5.97) background HIV prevalence, although the comparisons are limited because only two heterogeneous studies were included in higher prevalence settings.

Sexual risk factors in men showed greater differences in risk between levels of background prevalence than in women, although these differences by area were also not statistically significant. For example, the risk for HIV infection with more than 3 sex partners versus 0–2 was smaller in the lower HIV prevalence areas (OR = 2.68) than in the higher prevalence areas (OR = 3.79). Similarly, a history of STI showed smaller risk in the lower HIV prevalence areas (OR = 2.44) than in the higher prevalence areas (OR = 3.50). In contrast, HSV-2 history was associated with larger risk (OR = 7.60) in the lower HIV prevalence areas than in the higher prevalence areas (OR = 6.34).

There were no differences in individual HIV risk by background prevalence for being a FSW or being a male client of a FSW. Similarly, there were little differences in any of the risk categories for number of partners, being a FSW or her client, HSV-2 history or STI history by background rates of male circumcision (data not shown). Stratification by the Eastern and Southern African countries versus others also showed little difference in risk (results not shown).

### Comparison of high-risk and general populations

Table 1 provides a comparison of the summary ORs for various exposures by gender for both general and high-risk populations. All exposures were more prevalent among high-risk group controls than among general population controls, although these differences were not statistically significant. For example, 35% of high-risk group control women reported selling sex in contrast to only 3% among general population female controls. The OR for the association between HIV and paid sex was smaller in the general population than in the high-risk populations. For women, risks from HSV-2 were similar in general and high-risk populations, but there were too few studies to compare STI risk reliably. For men, there were no differences in HIV risk for being a client of a FSW between general and high-risk populations, but risks of HSV-2 or STI were larger in the general population than in the high-risk population.

**Table 1:** Summary ORs for general population and high-risk groups

Risk Factor and Gender		General Population				High-risk Groups			
Women	Men	No. of Studies	Exposure prev. in cases*	Exposure prev. in controls†	Summary OR (95% CI)	No. of Studies	Exposure prev. in cases*	Exposure prev. in controls†	Summary OR (95% CI)
Sex Partners: 3+ *vs* 0–2		12	33%	18%	3·64 [2·87–4·62]	1	42%	26%	2·04 [1·48–2·83]
Paid sex		9	7%	3%	2·29 [1·45–3·62]	6	50%	35%	2·42 [1·31–4·45]
STI		13	29%	12%	2·39 [2·01–2·84]	2	42%	48%	1·05 [0·68–1·63]
HSV-2		9	56%	30%	4·62 [2·85–7·47]	7	87%	56%	3·69 [2·56–5·33]
	Sex Partners: 3+ *vs* 0-2	6	65%	40%	3·15 [2·08–4·78]	.	.	.	·
	Paid sex	10	32%	18%	1·75 [1·30–2·36]	7	77%	69%	2·12 [1·43–3·15]
	STI	8	55%	25%	3·05 [2·22–4·20]	2	73%	57%	2·22 [0·95–5·16]
	HSV-2	7	60%	13%	6·97 [4·68–10·38]	2	62%	37%	2·75 [2·13–3·54]

* exposure prevalence in cases equals the number of HIV-positive individuals with exposure to the risk factor over the total number of HIV-positive individuals; † exposure prevalence in controls equals the number of HIV-negative individuals with exposure to the risk factor over the total number of HIV-negative individuals.

### Male-female prevalence ratios of risk factors among control populations

**Figure 7:**
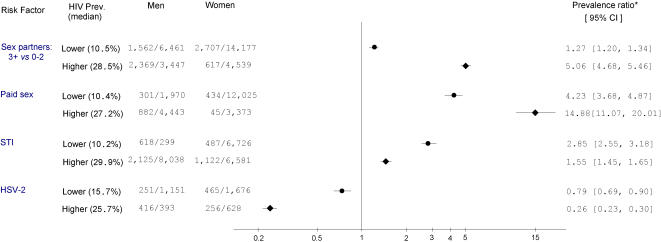
Male-female prevalence ratios for sexual risk factors among HIV-negative general populations, by lower and higher background HIV prevalence. Notes: * Prevalence ratio equals the rate of exposure among HIV-negative men over that of HIV-negative women.

Figure 7 presents the male-female ratio of the prevalence of selected risk factors (three or more sex partners versus 0–2 partners, paid sex, STI and HSV-2 history); stratified by lower or higher background prevalence of HIV. Interestingly, male-female prevalence ratios for 3 or more partners or history of paid sex were more extreme in higher prevalence settings than in lower prevalence settings. History of STI was more common among men, but the male-female prevalence ratio was larger in lower HIV prevalence settings. History of HSV-2 was more commonly reported among women than men, with a larger gap in lower background HIV prevalence areas.

### Publication bias, heterogeneity and confounding

**Figure 8:**
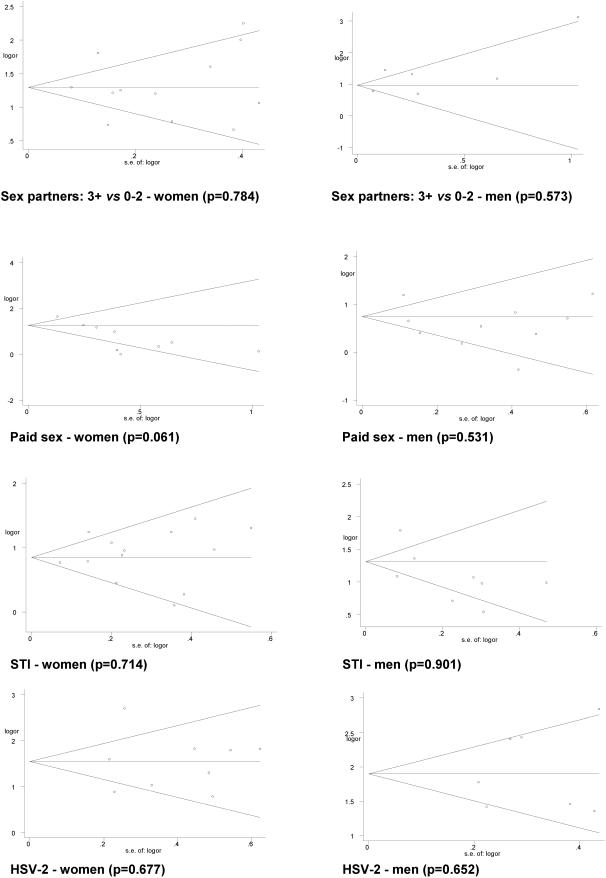
Begg's funnel plots with 95% CI for publication bias by risk factors (p<0.05 indicates a significant bias).

Funnel plots of the effects and the Egger rank correlation test did not show evidence of publication bias (Figure 8). There was no evidence suggesting that smaller studies with a larger standard error were more likely to report a positive association. Significant heterogeneity was found among studies in most of the risk factors. However, this heterogeneity fell when studies were stratified by population (general or high-risk), or by background HIV prevalence. Table 2 summarizes results for the 26 studies that adjusted for various possible confounding factors (as shown in Table S1). The adjusted odds ratios for most risk factors were similar to crude odds ratios, including a markedly higher excess risk of HIV infection from HSV-2 infection in males and females. Similarly, heterogeneity among individual studies was similar for the adjusted and crude odds ratios.

**Table 2:** Adjusted summary ORs for studies of the general population

Risk Factor and Gender		No. of studies	Adjusted summary OR (95% CI)
Women	Men		
Sex Partners: 3+ *vs* 0–2		.	.
Paid sex		2	0.70 [0.32–1.54]
STI		4	1.29 [0.92–1.80]
HSV-2		6	3.69 [2.78–4.89]
	Sex Partners: 3+ *vs* 0–2	.	.
	Paid sex	3	1.66 [1.31–2.12]
	STI	3	1.84 [1.33–2.56]
	HSV-2	5	4.96 [3.52–6.98]

Note: adjusted summary OR were calculated using fixed effects method of inverse variance [115].

## Discussion

### Key risk factors

This meta-analysis confirms that the key sexual risk factors for HIV infection in sub-Saharan African are a high number of lifetime sexual partners, engaging in paid sex, and having an STI-most notably HSV-2. We observed an approximately linear relationship between number of partners and HIV risk for both men and women. Indeed, one study [95] of male truck drivers found that risk was continuously increasing with numbers of sex partners even within the extreme ‘tail” of sexual behavior, with an OR of 4.0 in men reporting 10 or more partners (versus 1–9). Among HIV uninfected controls, the proportion of men reporting 3 or more sex partners (versus 0–2) was 40% vs. 18% among women (Table 1). Indeed, the prevalence of multi-partner sex among control women is larger than that that reported by women in general population surveys [119], suggesting that the control populations enrolled here were somewhat selected for higher risk. This would suggest that the true contribution of a high number of sexual partners to HIV risk might be greater than implied by the estimates shown here. Women tend to under-report ever having sold sex, and men may under-report paying for it [120]. Such misclassification would lead to our observed risks underestimating true risks. Finally, although we calculated crude risks, significant excess risks, especially for HSV-2, persisted in a sub-set of studies that adjusted for potentially confounding variables (Table 2).

### Consistency of sexual risk factors in advanced and early HIV epidemics

The key sexual risk factors appear to have changed little in magnitude during the last two decades. Indeed, stratification by advanced and newer (more recent) HIV epidemics (or by Eastern and Southern Africa versus other countries) show that most of the key risk factors are, if anything, more important in high prevalence settings (HSV-2 in men being the exception). It is perhaps counterintuitive that the strength of the association between sexual risk factors and HIV infection depends so little on background HIV prevalence or on time since the introduction of HIV infection. After all, when a high percentage of sex partners is HIV infected (and given that HIV is not highly transmissible per single sex act), one might expect otherwise. Four observations may help put these results into perspective.

First, male-female differences in multi-partner sex and paid sex were more extreme in higher prevalence than in lower prevalence settings (Figure 7). This is consistent with the idea that male sexual behavior (most significantly from paid sex) is important even in advanced epidemics. An earlier review [119] suggests that discrepancies between male and female reported partners in Africa arise, in part, from age mixing (i.e. older men having sex with younger women). If so, age mixing as a determinant of HIV transmission might be more extreme in higher prevalence settings than in lower prevalence settings, and deserves further study.

Second, the consistency of sexual risk factors for HIV over time, by advanced and early HIV epidemics, and the marked male-female differences among control groups emphasizes the importance of high-risk sexual behavior in transmission. HIV prevalence among FSWs in this study was much larger (28%) than among women with no history of paid sex (17%). The prevalence of HIV was 35% for men who reported sex with FSWs versus 21% for those who did not (data not shown). Small studies in Benin [121] and Ghana [122] find that even two decades after HIV was first introduced, most new infections in men are acquired from FSWs. Other findings suggest that high-risk sex also accounts for much of transmission to apparently low-risk women. UNAIDS [123] cites studies that found that nearly two-thirds of women in urban Zimbabwe, Durban and Soweto in South Africa reported only one lifetime partner and nearly four-fifths had abstained from sex until at least age 17 years. Yet 40% of women in these settings are HIV-positive. The most plausible explanation appears to be high-risk sexual behavior of their regular male partners, although sexual risk taking by male partners of low-risk women has been poorly studied.

Third, even when background HIV prevalence is high, a disproportionate proportion of transmission might be caused by a much smaller group of “super-spreaders”, i.e. highly infectious people with a high viral load and a high rate of partner change. Such heterogeneity in transmissibility is due to various factors, such as elevated genital tract HIV virus levels (thus raising transmissibility) during acute HIV infection [3], [7], and co-infection with other STIs, which increase genital tract HIV levels up to 10-fold [3]. In turn, an individual's probability of having sex with such a super-spreader would be determined by his/her own sexual behavior (or by their regular partner's behavior), more specifically by his/her rate of acquisition of new, notably high risk, sex partners. There is no plausible reason to assume that the biological features of super-spreaders or the crude correlates of an individual's probability of contacting such a super spreader (i.e. paid sex, multi-partner sex and STIs) would be relevant only in newer HIV epidemics. Indeed the higher mortality among these super-spreaders (due in perhaps to co-infections with tuberculosis and other factors) is one explanation for Uganda's remarkable decline in HIV prevalence in the general population from 1990 to 2003 [124].

Lastly, co-infections with other STIs have a role in facilitating transmission. We noted exceptionally high risks for HIV infection associated with HSV-2 infection among women and men, but this may reflect reverse causality, as HIV may facilitate diagnosis of HSV-2 by aggravating its clinical symptoms [4]. Nevertheless, a prospective study of sex workers in Kenya [125] suggested that prior HSV-2 is a key risk factor for acquiring HIV, arguing against such reverse causality. Other co-infections or agents have not been well explored in HIV epidemiology.

### Limitations of the study

The limitations of meta-analyses of observational studies in general [126] also apply here. The majority of studies were cross-sectional, i.e. exposures and HIV status had been determined at the same time. Also we were limited in our choice of risk factors. For instance, we could not explore concurrency, volume of sexual contacts, or duration of selling or buying sex, all of which might be highly relevant in HIV chains of transmission [7]. However, crude determinants, such as lifetime number of partners, are likely to be strongly correlated with these more refined measures. Misclassification of exposures such as sex partners, paid sex and STI may be present in all studies based on self-reported risk factors. Fortunately, bias for HSV-2 is unlikely as it was based on clinical examination, perhaps contributing to its strength as a risk factor for HIV infection. Misclassification of the outcome, i.e. HIV status is unlikely as nearly all studies performed two ELISA tests. Studies from Africa are under-reported in medical publication databases [127]–[28], although our statistical tests for missing publications did not suggest this biased our conclusions.

Our primary objective was to look at *trends* in risks and *differences* between high and low prevalence. While it is well recognized that the above discussed biases, as well as individual study quality, may have a large impact on summary odds ratios in meta analyses [6], these flaws are less likely to bias trends in odds ratios rather than summary odds ratios themselves. There is no reason to believe that study quality changed over time in ways that might invalidate our conclusions. Certainly, the underlying heterogeneity of risks from each study, while wholly expected, reduces the confidence in the validity of some of our summary estimates of risk. Yet, this heterogeneity should not differ over time or between settings with lower and higher background HIV prevalence. Similarly, some of this heterogeneity may reflect lack of adjustment for relevant confounders. Regrettably, adjustment for potential confounding factors is not straightforward in STI epidemiology, given that various causal pathways are possible [129] and most studies did not record, or adjust for, all potential confounders. Where such adjustment was done, it did not appear to change our overall results, which would seem logical given that these confounders would also not be expected to vary differ over time or between settings with lower and higher background HIV prevalence. Indeed, for the 11 HSV-2 studies that permitted adjustment, the (lack of) trends in adjusted risks were similar to those in crude risks (results not shown).

### Implications

Our study results have four key implications. First, there is an urgent need to establish standardized epidemiological studies that can reliably quantify individual differences in risk, understand the chain of HIV transmission, and track changes in both over time (including in response to control programs). Our search strategy identified marked variation in study quality- implying that standardized studies in different populations are required. For example, studies could assess if infected individuals were one, two or several contacts away from a putatively infected vulnerable group contact, and to what degree co-factors of transmission such as STIs are important. Such studies are particularly important to explore possible reasons for the rapid-and largely unexplained-growth of HIV in the general population in Eastern and Southern Africa. Second, there is a need for a careful re-examination of the UNAIDS definition of ‘generalized’ epidemic. If the key sexual risk factors in advanced epidemics are similar to those in newer (recent) ones, then this concept is relevant only as a convenient classification of HIV prevalence levels, but otherwise lacks any relevance with respect to underlying epidemiological processes. Third, the study appears to contradict the WHO/UNAIDS expert group recommendations [130] that the population impact of STI treatment on HIV incidence is likely to be smaller in advanced HIV epidemics where rates of curable STIs are either declining or low. Fourth, and most importantly, our results suggest that the preventative programs in much of Africa should focus on interventions among vulnerable groups irrespective of the population levels of infection [7]–[8].

## Supporting Information

Table S1 (0.08 MB XLS)

## References

[1] UNAIDS (2006). AIDS Epidemic Update..

[2] Cote AM, Sobela F, Dzokoto A, Nzambi K, Samoah-Adu C (2004). Transactional sex is the driving force in the dynamics of HIV in Accra, Ghana.. AIDS.

[3] Cohen MS, Hoffman IF, Royce RA, Kazembe P, Dyer JR (1997). Reduction of concentration of HIV-1 in semen after treatment of urethritis: implications for prevention of sexual transmission of HIV-1. AIDSCAP Malawi Research Group.. Lancet.

[4] Freeman EE, Weiss HA, Glynn JR, Cross PL, Whitworth JA (2006). Herpes simplex virus 2 infection increases HIV acquisition in men and women: systematic review and meta-analysis of longitudinal studies.. AIDS.

[5] Weiss HA, Quigley MA, Hayes RJ (2000). Male circumcision and risk of HIV infection in sub-Saharan Africa: a systematic review and meta-analysis.. AIDS.

[6] Siegfried N, Muller M, Deeks J, Volmink J, Egger M (2005). HIV and male circumcision–a systematic review with assessment of the quality of studies.. Lancet Infect Dis.

[7] Nagelkerke NJ, Jha P, de Vlas SJ, Korenromp EL, Moses S (2002). Modelling HIV/AIDS epidemics in Botswana and India: impact of interventions to prevent transmission.. Bull World Health Organ.

[8] Jha P, Nagelkerke JD, Ngugi EN, Prasada Rao JV, Willbond B (2001). Reducing HIV transmission in developing countries.. Science.

[9] Hearst N, Mandel JS (1997). A research agenda for AIDS prevention in the developing world.. AIDS.

[10] Auvert B, Taljaard D, Lagarde E, Sobngwi-Tambekou J, Sitta R (2005). Randomized, controlled intervention trial of male circumcision for reduction of HIV infection risk: the ANRS 1265 Trial.. PLoS Med.

[11] Allen S, Lindan C, Serufilira A, Van de PP, Rundle AC (1991). Human immunodeficiency virus infection in urban Rwanda. Demographic and behavioral correlates in a representative sample of childbearing women.. JAMA.

[12] Lindan C, Allen S, Carael M, Nsengumuremyi F, Van de PP (1991). Knowledge, attitudes, and perceived risk of AIDS among urban Rwandan women: relationship to HIV infection and behavior change.. AIDS.

[13] Bassett MT, Latif AS, Katzenstein DA, Emmanuel JC (1992). Sexual behavior and risk factors for HIV infection in a group of male factory workers who donated blood in Harare, Zimbabwe.. J Acquir Immune Defic Syndr.

[14] Berkley SF, Widy-Wirski R, Okware SI, Downing R, Linnan MJ (1989). Risk factors associated with HIV infection in Uganda.. J Infect Dis.

[15] Miotti PG, Dallabetta G, Ndovi E, Liomba G, Saah AJ (1990). HIV-1 and pregnant women: associated factors, prevalence, estimate of incidence and role in fetal wastage in central Africa.. AIDS.

[16] Chao A, Bulterys M, Musanganire F, Habimana P, Nawrocki P (1994). Risk factors associated with prevalent HIV-1 infection among pregnant women in Rwanda. National University of Rwanda-Johns Hopkins University AIDS Research Team.. Int J Epidemiol.

[17] Hunter DJ, Maggwa BN, Mati JK, Tukei PM, Mbugua S (1994). Sexual behavior, sexually transmitted diseases, male circumcision and risk of HIV infection among women in Nairobi, Kenya.. AIDS.

[18] Serwadda D, Wawer MJ, Musgrave SD, Sewankambo NK, Kaplan JE (1992). HIV risk factors in three geographic strata of rural Rakai District, Uganda.. AIDS.

[19] Ndinya-Achola JO, Ghee AE, Kihara AN, Krone MR, Plummer FA (1997). High HIV prevalence, low condom use and gender differences in sexual behaviour among patients with STD-related complaints at a Nairobi primary health care clinic.. Int J STD AIDS.

[20] Nunn AJ, Wagner HU, Okongo JM, Malamba SS, Kengeya-Kayondo JF (1996). HIV-1 infection in a Ugandan town on the trans-African highway: prevalence and risk factors.. Int J STD AIDS.

[21] Carpenter LM, Kamali A, Payne M, Kiwuuwa S, Kintu P (2002). Independent effects of reported sexually transmitted infections and sexual behavior on HIV-1 prevalence among adult women, men, and teenagers in rural Uganda.. J Acquir Immune Defic Syndr.

[22] Quigley M, Munguti K, Grosskurth H, Todd J, Mosha F (1997). Sexual behaviour patterns and other risk factors for HIV infection in rural Tanzania: a case-control study.. AIDS.

[23] Bulterys M, Chao A, Habimana P, Dushimimana A, Nawrocki P (1994). Incident HIV-1 infection in a cohort of young women in Butare, Rwanda.. AIDS.

[24] Siziya S, Hakim JG (1996). Differential human immunodeficiency virus risk factors among female general nurses, nurse midwives and office workers/teachers in Zambia.. Cent Afr J Med.

[25] Cossa HA, Gloyd S, Vaz RG, Folgosa E, Simbine E (1994). Syphilis and HIV infection among displaced pregnant women in rural Mozambique.. Int J STD AIDS.

[26] del Mar Pujades RM, Obasi A, Mosha F, Todd J, Brown D (2002). Herpes simplex virus type 2 infection increases HIV incidence: a prospective study in rural Tanzania.. AIDS.

[27] Todd J, Grosskurth H, Changalucha J, Obasi A, Mosha F (2006). Risk factors influencing HIV infection incidence in a rural African population: a nested case-control study.. J Infect Dis.

[28] Kapiga SH, Lyamuya EF, Lwihula GK, Hunter DJ (1998). The incidence of HIV infection among women using family planning methods in Dar es Salaam, Tanzania.. AIDS.

[29] Quigley MA, Morgan D, Malamba SS, Mayanja B, Okongo MJ (2000). Case-control study of risk factors for incident HIV infection in rural Uganda.. J Acquir Immune Defic Syndr.

[30] Bassett MT, McFarland WC, Ray S, Mbizvo MT, Machekano R (1996). Risk factors for HIV infection at enrollment in an urban male factory cohort in Harare, Zimbabwe.. J Acquir Immune Defic Syndr Hum Retrovirol.

[31] Kelly R, Kiwanuka N, Wawer MJ, Serwadda D, Sewankambo NK (1999). Age of male circumcision and risk of prevalent HIV infection in rural Uganda.. AIDS.

[32] Mbizvo MT, Mashu A, Chipato T, Makura E, Bopoto R (1996). Trends in HIV-1 and HIV-2 prevalence and risk factors in pregnant women in Harare, Zimbabwe.. Cent Afr J Med.

[33] Aseffa A, Ishak A, Stevens R, Fergussen E, Giles M (1998). Prevalence of HIV, syphilis and genital chlamydial infection among women in north-west Ethiopia.. Epidemiol Infect.

[34] McFarland W, Gwanzura L, Bassett MT, Machekano R, Latif AS (1999). Prevalence and incidence of herpes simplex virus type 2 infection among male Zimbabwean factory workers.. J Infect Dis.

[35] Tengia-Kessy A, Msamanga GI, Moshiro CS (1998). Assessment of behavioural risk factors associated with HIV infection among youth in Moshi rural district, Tanzania.. East Afr Med J.

[36] Kapiga SH, Shao JF, Lwihula GK, Hunter DJ (1994). Risk factors for HIV infection among women in Dar-es-Salaam, Tanzania.. J Acquir Immune Defic Syndr.

[37] Hargreaves JR (2002). Socioeconomic status and risk of HIV infection in an urban population in Kenya.. Trop Med Int Health.

[38] Mihret W, Rinke de Wit TF, Petros B, Mekonnen Y, Tsegaye A (2002). Herpes simplex virus type 2 seropositivity among urban adults in Africa: results from two cross-sectional surveys in Addis Ababa, Ethiopia.. Sex Transm Dis.

[39] Kumwenda NI, Taha TE, Hoover DR, Markakis D, Liomba GN (2002). Three surveys of HIV-1 prevalence and risk factors among men working at a sugar estate in Malawi.. Sex Transm Dis.

[40] Lagarde E, Schim van der LM, Enel C, Holmgren B, Dray-Spira R (2003). Mobility and the spread of human immunodeficiency virus into rural areas of West Africa.. Int J Epidemiol.

[41] Mbopi-Keou FX, Gresenguet G, Mayaud P, Weiss HA, Gopal R (1999). Genital herpes simplex virus type 2 shedding is increased in HIV-infected women in Africa.. AIDS.

[42] Sutcliffe S, Taha TE, Kumwenda NI, Taylor E, Liomba GN (2002). HIV-1 prevalence and herpes simplex virus 2, hepatitis C virus, and hepatitis B virus infections among male workers at a sugar estate in Malawi.. J Acquir Immune Defic Syndr.

[43] Auvert B, Ballard R, Campbell C, Carael M, Carton M (2001). HIV infection among youth in a South African mining town is associated with herpes simplex virus-2 seropositivity and sexual behaviour.. AIDS.

[44] Coffee MP, Garnett GP, Mlilo M, Voeten HA, Chandiwana S (2005). Patterns of movement and risk of HIV infection in rural Zimbabwe.. J Infect Dis.

[45] Lurie MN, Williams BG, Zuma K, Mkaya-Mwamburi D, Garnett G (2003). The impact of migration on HIV-1 transmission in South Africa: a study of migrant and nonmigrant men and their partners.. Sex Transm Dis.

[46] Msuya SE, Mbizvo E, Stray-Pedersen B, Sundby J, Sam NE (2002). Reproductive tract infections and the risk of HIV among women in Moshi, Tanzania.. Acta Obstet Gynecol Scand.

[47] Mbizvo EM, Msuya SE, Stray-Pedersen B, Sundby J, Chirenje MZ (2001). HIV seroprevalence and its associations with the other reproductive tract infections in asymptomatic women in Harare, Zimbabwe.. Int J STD AIDS.

[48] Mbizvo EM, Msuya SE, Stray-Pedersen B, Chirenje MZ, Munjoma M (2002). Association of herpes simplex virus type 2 with the human immunodeficiency virus among urban women in Zimbabwe.. Int J STD AIDS.

[49] Cowan FM, Langhaug LF, Hargrove JW, Jaffar S, Mhuriyengwe L (2005). Is sexual contact with sex workers important in driving the HIV epidemic among men in rural Zimbabwe?. J Acquir Immune Defic Syndr.

[50] Lagarde E, Congo Z, Meda N, Baya B, Yaro S (2004). Epidemiology of HIV infection in urban Burkina Faso.. Int J STD AIDS.

[51] Nyambi P, Zekeng L, Kenfack H, Tongo M, Nanfack A (2002). HIV infection in rural villages of Cameroon.. J Acquir Immune Defic Syndr.

[52] Clift S, Anemona A, Watson-Jones D, Kanga Z, Ndeki L (2003). Variations of HIV and STI prevalences within communities neighbouring new goldmines in Tanzania: importance for intervention design.. Sex Transm Infect.

[53] Pettifor AE, van der SA, Dunbar MS, Shiboski SC, Padian NS (2004). Early age of first sex: a risk factor for HIV infection among women in Zimbabwe.. AIDS.

[54] Stringer EM, Sinkala M, Kumwenda R, Chapman V, Mwale A (2004). Personal risk perception, HIV knowledge and risk avoidance behavior, and their relationships to actual HIV serostatus in an urban African obstetric population.. J Acquir Immune Defic Syndr.

[55] Sagay AS, Kapiga SH, Imade GE, Sankale JL, Idoko J (2005). HIV infection among pregnant women in Nigeria.. Int J Gynaecol Obstet.

[56] Zuma K, Gouws E, Williams B, Lurie M (2003). Risk factors for HIV infection among women in Carletonville, South Africa: migration, demography and sexually transmitted diseases.. Int J STD AIDS.

[57] Barongo LR, Borgdorff MW, Mosha FF, Nicoll A, Grosskurth H (1992). The epidemiology of HIV-1 infection in urban areas, roadside settlements and rural villages in Mwanza Region, Tanzania.. AIDS.

[58] Kelly RJ, Gray RH, Sewankambo NK, Serwadda D, Wabwire-Mangen F (2003). Age differences in sexual partners and risk of HIV-1 infection in rural Uganda.. J Acquir Immune Defic Syndr.

[59] Mnyika KS, Klepp KI, Kvale G, Ole-King'ori N (1996). Risk factors for HIV-1 infection among women in the Arusha region of Tanzania.. J Acquir Immune Defic Syndr Hum Retrovirol.

[60] Simonsen JN, Cameron DW, Gakinya MN, Ndinya-Achola JO, D'Costa LJ (1988). Human immunodeficiency virus infection among men with sexually transmitted diseases. Experience from a center in Africa.. N Engl J Med.

[61] Nsubuga P, Mugerwa R, Nsibambi J, Sewankambo N, Katabira E (1990). The association of genital ulcer disease and HIV infection at a dermatology-STD clinic in Uganda.. J Acquir Immune Defic Syndr.

[62] Nzila N, Laga M, Thiam MA, Mayimona K, Edidi B (1991). HIV and other sexually transmitted diseases among female prostitutes in Kinshasa.. AIDS.

[63] Plourde PJ, Plummer FA, Pepin J, Agoki E, Moss G (1992). Human immunodeficiency virus type 1 infection in women attending a sexually transmitted diseases clinic in Kenya.. J Infect Dis.

[64] Seed J, Allen S, Mertens T, Hudes E, Serufilira A (1995). Male circumcision, sexually transmitted disease, and risk of HIV.. J Acquir Immune Defic Syndr Hum Retrovirol.

[65] Diallo MO, Ackah AN, Lafontaine MF, Doorly R, Roux R (1992). HIV-1 and HIV-2 infections in men attending sexually transmitted disease clinics in Abidjan, Cote d'Ivoire.. AIDS.

[66] Bwayo J, Plummer F, Omari M, Mutere A, Moses S (1994). Human immunodeficiency virus infection in long-distance truck drivers in east Africa.. Arch Intern Med.

[67] Sassan-Morokro M, Greenberg AE, Coulibaly IM, Coulibaly D, Sidibe K (1996). High rates of sexual contact with female sex workers, sexually transmitted diseases, and condom neglect among HIV-infected and uninfected men with tuberculosis in Abidjan, Cote d'Ivoire.. J Acquir Immune Defic Syndr Hum Retrovirol.

[68] Marcelin AG, Grandadam M, Flandre P, Nicand E, Milliancourt C (2002). Kaposi's sarcoma herpesvirus and HIV-1 seroprevalences in prostitutes in Djibouti.. J Med Virol.

[69] Langeland N, Haarr L, Mhalu F (1998). Prevalence of HSV-2 antibodies among STD clinic patients in Tanzania.. Int J STD AIDS.

[70] Meda N, Ledru S, Fofana M, Lankoande S, Soula G (1995). Sexually transmitted diseases and human immunodeficiency virus infection among women with genital infections in Burkina Faso.. Int J STD AIDS.

[71] Chen CY, Ballard RC, Beck-Sague CM, Dangor Y, Radebe F (2000). Human immunodeficiency virus infection and genital ulcer disease in South Africa: the herpetic connection.. Sex Transm Dis.

[72] MacDonald KS, Malonza I, Chen DK, Nagelkerke NJ, Nasio JM (2001). Vitamin A and risk of HIV-1 seroconversion among Kenyan men with genital ulcers.. AIDS.

[73] Mbopi-Keou FX, Gresenguet G, Mayaud P, Weiss HA, Gopal R (2000). Interactions between herpes simplex virus type 2 and human immunodeficiency virus type 1 infection in African women: opportunities for intervention.. J Infect Dis.

[74] Kapiga SH, Sam NE, Shao JF, Renjifo B, Masenga EJ (2002). HIV-1 epidemic among female bar and hotel workers in northern Tanzania: risk factors and opportunities for prevention.. J Acquir Immune Defic Syndr.

[75] Kapiga SH, Sam NE, Shao JF, Masenga EJ, Renjifo B (2003). Herpes simplex virus type 2 infection among bar and hotel workers in northern Tanzania: prevalence and risk factors.. Sex Transm Dis.

[76] Riedner G, Rusizoka M, Hoffmann O, Nichombe F, Lyamuya E (2003). Baseline survey of sexually transmitted infections in a cohort of female bar workers in Mbeya Region, Tanzania.. Sex Transm Infect.

[77] Ao TT, Sam NE, Masenga EJ, Seage GR III, Kapiga SH (2006). Human immunodeficiency virus type 1 among bar and hotel workers in northern Tanzania: the role of alcohol, sexual behavior, and herpes simplex virus type 2.. Sex Transm Dis.

[78] ter Meulen J, Mgaya HN, Chang-Claude J, Luande J, Mtiro H (1992). Risk factors for HIV infection in gynaecological inpatients in Dar es Salaam, Tanzania, 1988–1990.. East Afr Med J.

[79] Maggwa BN, Hunter DJ, Mbugua S, Tukei P, Mati JK (1993). The relationship between HIV infection and cervical intraepithelial neoplasia among women attending two family planning clinics in Nairobi, Kenya.. AIDS.

[80] Taha TE, Hoover DR, Dallabetta GA, Kumwenda NI, Mtimavalye LA (1998). Bacterial vaginosis and disturbances of vaginal flora: association with increased acquisition of HIV.. AIDS.

[81] Smith J, Nalagoda F, Wawer MJ, Serwadda D, Sewankambo N (1999). Education attainment as a predictor of HIV risk in rural Uganda: results from a population-based study.. Int J STD AIDS.

[82] Zaba BW, Carpenter LM, Boerma JT, Gregson S, Nakiyingi J (2000). Adjusting ante-natal clinic data for improved estimates of HIV prevalence among women in sub-Saharan Africa.. AIDS.

[83] Kapiga SH, Lyamuya EF, Vuylsteke B, Spiegelman D, Larsen U (2000). Risk factors for HIV-1 seroprevalence among family planning clients in Dar es Salaam, Tanzania.. Afr J Reprod Health.

[84] Boerma JT, Gregson S, Nyamukapa C, Urassa M (2003). Understanding the uneven spread of HIV within Africa: comparative study of biologic, behavioral, and contextual factors in rural populations in Tanzania and Zimbabwe.. Sex Transm Dis.

[85] Gwanzura L, McFarland W, Alexander D, Burke RL, Katzenstein D (1998). Association between human immunodeficiency virus and herpes simplex virus type 2 seropositivity among male factory workers in Zimbabwe.. J Infect Dis.

[86] Simooya OO, Sanjobo NE, Kaetano L, Sijumbila G, Munkonze FH (2001). ‘Behind walls’: a study of HIV risk behaviours and seroprevalence in prisons in Zambia.. AIDS.

[87] Leroy V, De CA, Ladner J, Bogaerts J, Van de PP (1995). Should screening of genital infections be part of antenatal care in areas of high HIV prevalence? A prospective cohort study from Kigali, Rwanda, 1992–1993. The Pregnancy and HIV (EGE) Group.. Genitourin Med.

[88] Petry KU, Kingu H (1996). HIV infection among pregnant women in Lindi, Tanzania, 1989–1993.. Int J STD AIDS.

[89] Msuya SE, Mbizvo E, Hussain A, Sam NE, Jeansson S (2003). Seroprevalence and correlates of herpes simplex virus type 2 among urban Tanzanian women.. Sex Transm Dis.

[90] Van de PP, Carael M, Nzaramba D, Zissis G, Kayihigi J (1987). Risk factors for HIV seropositivity in selected urban-based Rwandese adults.. AIDS.

[91] Hira SK, Kamanga J, Macuacua R, Mwansa N, Cruess DF (1990). Genital ulcers and male circumcision as risk factors for acquiring HIV-1 in Zambia.. J Infect Dis.

[92] Grosskurth H, Mosha F, Todd J, Senkoro K, Newell J (1995). A community trial of the impact of improved sexually transmitted disease treatment on the HIV epidemic in rural Tanzania: 2. Baseline survey results.. AIDS.

[93] Urassa M, Todd J, Boerma JT, Hayes R, Isingo R (1997). Male circumcision and susceptibility to HIV infection among men in Tanzania.. AIDS.

[94] Sentjens RE, Sisay Y, Vrielink H, Kebede D, Ader HJ (2002). Prevalence of and risk factors for HIV infection in blood donors and various population subgroups in Ethiopia.. Epidemiol Infect.

[95] Carswell JW, Lloyd G, Howells J (1989). Prevalence of HIV-1 in east African lorry drivers.. AIDS.

[96] Thior I, Diouf G, Diaw IK, Sarr AD, Hsieh CC (1997). Sexually transmitted diseases and risk of HIV infection in men attending a sexually transmitted diseases clinic in Dakar, Senegal.. Afr J Reprod Health.

[97] Lankoande S, Meda N, Sangare L, Compaore IP, Catraye J (1998). Prevalence and risk of HIV infection among female sex workers in Burkina Faso.. Int J STD AIDS.

[98] Mann JM, Nzilambi N, Piot P, Bosenge N, Kalala M (1988). HIV infection and associated risk factors in female prostitutes in Kinshasa, Zaire.. AIDS.

[99] Ramjee G, Williams B, Gouws E, Van DE, De DB (2005). The impact of incident and prevalent herpes simplex virus-2 infection on the incidence of HIV-1 infection among commercial sex workers in South Africa.. J Acquir Immune Defic Syndr.

[100] Nagot N, Ouedraogo A, Ouangre A, Cartoux M, Defer MC (2005). Is sexually transmitted infection management among sex workers still able to mitigate the spread of HIV infection in West Africa?. J Acquir Immune Defic Syndr.

[101] Bogaerts J, Kestens L, Van DE, Tello WM, Akingeneye J (1998). Genital ulcers in a primary health clinic in Rwanda: impact of HIV infection on diagnosis and ulcer healing (1986–1992).. Int J STD AIDS.

[102] Bwayo JJ, Omari AM, Mutere AN, Jaoko W, Sekkade-Kigondu C (1991). Long distance truck-drivers: 1. Prevalence of sexually transmitted diseases (STDs).. East Afr Med J.

[103] Carael M, Van de Perre PH, Lepage PH, Allen S, Nsengumuremyi F (1988). Human immunodeficiency virus transmission among heterosexual couples in Central Africa.. AIDS.

[104] Ryder RW, Ndilu M, Hassig SE, Kamenga M, Sequeira D (1990). Heterosexual transmission of HIV-1 among employees and their spouses at two large businesses in Zaire.. AIDS.

[105] Lurie MN, Williams BG, Zuma K, Mkaya-Mwamburi D, Garnett GP (2003). Who infects whom? HIV-1 concordance and discordance among migrant and non-migrant couples in South Africa.. AIDS.

[106] Hudson CP, Hennis AJ, Kataaha P, Lloyd G, Moore AT (1988). Risk factors for the spread of AIDS in rural Africa: evidence from a comparative seroepidemiological survey of AIDS, hepatitis B and syphilis in southwestern Uganda.. AIDS.

[107] Greenblatt RM, Lukehart SA, Plummer FA, Quinn TC, Critchlow CW (1988). Genital ulceration as a risk factor for human immunodeficiency virus infection.. AIDS.

[108] McCarthy MC, Hyams KC, el-Tigani el-Hag A, el-Dabi MA, el-Sadig el-Tayeb M (1989). HIV-1 and hepatitis B transmission in Sudan.. AIDS.

[109] Pison G, Le GB, Lagarde E, Enel C, Seck C (1993). Seasonal migration: a risk factor for HIV infection in rural Senegal.. J Acquir Immune Defic Syndr.

[110] McCarthy MC, Khalid IO, El TA (1995). HIV-1 infection in Juba, southern Sudan.. J Med Virol.

[111] Sinei SK, Fortney JA, Kigondu CS, Feldblum PJ, Kuyoh M (1996). Contraceptive use and HIV infection in Kenyan family planning clinic attenders.. Int J STD AIDS.

[112] Gomo E, Chibatamoto PP, Chandiwana SK, Sabeta CT (1997). Risk factors for HIV infection in a rural cohort in Zimbabwe: a pilot study.. Cent Afr J Med.

[113] Obisesan KA, Olaleye OD, Adeyemo AA (1997). The increasing prevalence of HIV-1 and HIV-2 infections in a low-risk antenatal population in south west Nigeria.. Int J Gynaecol Obstet.

[114] WHO/UNAIDS (2002). HIV Simple/Rapid Assays: Operational Characteristics, Report 13..

[115] Sutton AJ (2000). Methods for Meta-analysis in Medical Research..

[116] Seber GAF, Lee AJ (2003). Linear Regression Analysis. 2nd ed..

[117] Breslow NE, Day NE (1980). Statistical Methods in Cancer Research. Vol I: The Analysis of Case-Control Studies. IARC Sci Publ No 32..

[118] Egger M, Smith GD, Altman DG (2001). Systematic reviews in health care: meta-analysis in context. 2nd ed..

[119] Wellings K, Collumbien M, Slaymaker E, Singh S, Hodges Z (2006). Sexual behaviour in context: a global perspective.. Lancet.

[120] Cleland J, Boerma JT, Carael M, Weir SS (2004). Monitoring sexual behaviour in general populations: a synthesis of lessons of the past decade.. Sex Transm Infect.

[121] Alary M, Mukenge-Tshibaka L, Bernier F, Geraldo N, Lowndes CM (2002). Decline in the prevalence of HIV and sexually transmitted diseases among female sex workers in Cotonou, Benin, 1993–1999.. AIDS.

[122] Samoah-Adu C, Khonde N, Avorkliah M, Bekoe V, Alary M (2001). HIV infection among sex workers in Accra: need to target new recruits entering the trade.. J Acquir Immune Defic Syndr.

[123] UNAIDS (2005). AIDS Epidemic Update: Special Section on HIV Prevention..

[124] Wawer MJ, Gray R, Serwadda D, Namukwaya Z, Makumbi F (2005). Declines in HIV prevalence in Uganda: not as simple as ABC.. Twelfth conference on retroviruses and opportunistic infections, Boston: No 27LB.

[125] Kaul R, Kimani J, Nagelkerke NJ, Fonck K, Ngugi EN (2004). Monthly antibiotic chemoprophylaxis and incidence of sexually transmitted infections and HIV-1 infection in Kenyan sex workers: a randomized controlled trial.. JAMA.

[126] Stroup DF, Berlin JA, Morton SC, Olkin I, Williamson GD (2000). Meta-analysis of observational studies in epidemiology: a proposal for reporting. Meta-analysis Of Observational Studies in Epidemiology (MOOSE) group.. JAMA.

[127] Egger M, Davey-Smith G (1998). Meta-analysis: Bias in location and selection of studies.. BMJ.

[128] Siegfried N, Clarke M, Volmink J (2005). Randomised controlled trials in Africa of HIV and AIDS: descriptive study and spatial distribution.. BMJ.

[129] Korenromp EL, de Vlass SJ, Nagelkerke NJ, Habbema JD (2001). Estimating the magnitude of STD cofactor effects on HIV transmission: how well can it be done?. Sex Transm Dis..

[130] WHO/UNAIDS Consultation on STI interventions for preventing HIV: appraisal of the evidence.. http://data.unaids.org/Publications/RC-pub01/JC300-ConsultSTD.

